# Pumping and sliding of droplets steered by a hydrogel pattern for atmospheric water harvesting

**DOI:** 10.1093/nsr/nwad334

**Published:** 2023-12-28

**Authors:** Wei Zhang, Qinghua Ji, Gong Zhang, Zhenao Gu, Haozhi Wang, Chengzhi Hu, Huijuan Liu, Zhiyong Jason Ren, Jiuhui Qu

**Affiliations:** Center for Water and Ecology, State Key Joint Laboratory of Environment Simulation and Pollution Control, School of Environment, Tsinghua University, Beijing 100084, China; Key Laboratory of Drinking Water Science and Technology, Research Center for Eco-Environmental Sciences, Chinese Academy of Sciences, Beijing 100085, China; University of Chinese Academy of Sciences, Beijing 100049, China; Center for Water and Ecology, State Key Joint Laboratory of Environment Simulation and Pollution Control, School of Environment, Tsinghua University, Beijing 100084, China; Center for Water and Ecology, State Key Joint Laboratory of Environment Simulation and Pollution Control, School of Environment, Tsinghua University, Beijing 100084, China; Key Laboratory of Drinking Water Science and Technology, Research Center for Eco-Environmental Sciences, Chinese Academy of Sciences, Beijing 100085, China; University of Chinese Academy of Sciences, Beijing 100049, China; Joint School of National University of Singapore and Tianjin University, International Campus of Tianjin University, Fuzhou 350207, China; Key Laboratory of Advanced Ceramics and Machining Technology (Ministry of Education), School of Materials Science and Engineering, Tianjin University, Tianjin 300072, China; Key Laboratory of Drinking Water Science and Technology, Research Center for Eco-Environmental Sciences, Chinese Academy of Sciences, Beijing 100085, China; University of Chinese Academy of Sciences, Beijing 100049, China; Center for Water and Ecology, State Key Joint Laboratory of Environment Simulation and Pollution Control, School of Environment, Tsinghua University, Beijing 100084, China; Department of Civil and Environmental Engineering and Andlinger Center for Energy and the Environment, Princeton University, Princeton, NJ 08544, USA; Center for Water and Ecology, State Key Joint Laboratory of Environment Simulation and Pollution Control, School of Environment, Tsinghua University, Beijing 100084, China; Key Laboratory of Drinking Water Science and Technology, Research Center for Eco-Environmental Sciences, Chinese Academy of Sciences, Beijing 100085, China; University of Chinese Academy of Sciences, Beijing 100049, China

**Keywords:** atmospheric water harvesting, droplet manipulation, bio-inspired, condensation

## Abstract

Atmospheric water harvesting is an emerging strategy for decentralized and potable water supplies. However, water nucleation and microdroplet coalescence on condensing surfaces often result in surface flooding owing to the lack of a sufficient directional driving force for shedding. Herein, inspired by the fascinating properties of lizards and catfish, we present a condensing surface with engineered hydrogel patterns that enable rapid and sustainable water harvesting through the directional pumping and drag-reduced sliding of water droplets. The movement of microscale condensed droplets is synergistically driven by the surface energy gradient and difference in Laplace pressure induced by the arch hydrogel patterns. Meanwhile, the superhydrophilic hydrogel surface can strongly bond inner-layer water molecules to form a lubricant film that reduces drag and facilitates the sliding of droplets off the condensing surface. Thus, this strategy is promising for various water purification techniques based on liquid–vapor phase-change processes.

## Teaser

Hydrogel fibers were proposed to integrate merits of lizard and catfish, creating surface energy gradients and Laplace pressure difference to directionally steer the droplets for enhanced atmospheric water harvesting.

## INTRODUCTION

Atmospheric water harvesting can potentially address the global water crisis, especially in areas lacking clean water sources [1]. Condensation is a fundamental step for water-harvesting systems and is ubiquitous in water purification and energy conversion [2]. It is an energy-intensive exothermic phase-change process involving two key steps: droplet growth and shedding. Generally, nanoscale droplets preferentially form on hydrophilic sites, which release latent heat, and grow through random cohesion between neighboring droplets [2,3]. However, droplet overgrowth and heat release often block condensing sites and choke sustainable condensation. Thus, condensing site regeneration is vital for water harvesting, which relies on fast droplet growth and transport [2,4,5]. Unfortunately, surface design strategies suffer from an intrinsic trade-off between these processes [4]. Conventional strategies mainly rely on (dis)ordered microstructures and superhydrophobic/philic treatment to regulate droplet movement and facilitate condensation. However, the condensing sites are sacrificed due to hydrophobicity [4,6–9]. Moreover, droplets must reach a critical size through coalescence to gain sufficient energy for movement, which introduces uncertainties in the trajectory. During droplet shedding, a lack of engineered pathways causes continued growth through random cohesions with smaller droplets. Amplified contact angle hysteresis, especially on the microscale, results in an insufficient driving force and condensing site flooding [10]. Therefore, surfaces capable of directional transport of micrometer droplets and possessing regenerating condensing sites are highly desirable for sustainable condensation.

**Figure 1.**
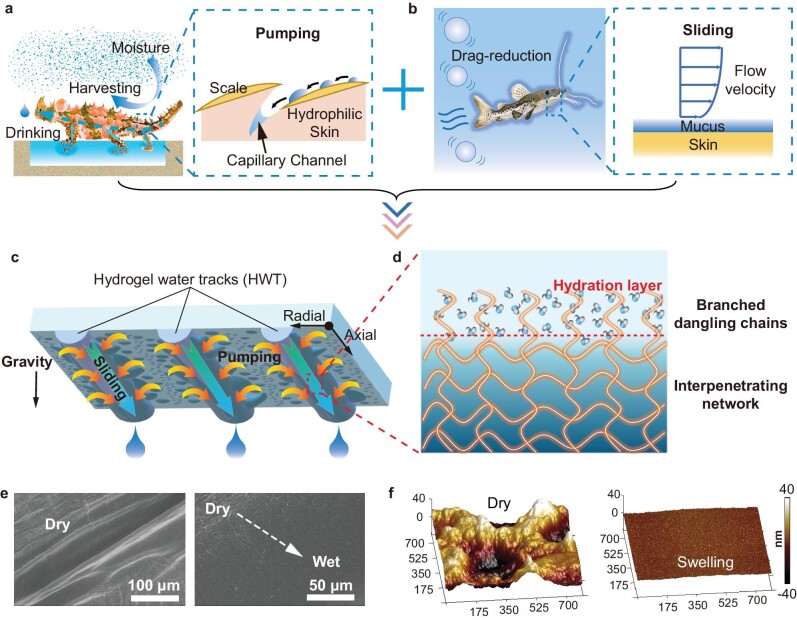
Biomimetic HWT design for rapid water harvesting. (a) Schematic of continuous water capture and directional water movement for drinking by moisture-harvesting lizards enabled by the capillary channels between the scale (side length is ∼1 mm). (b) Schematic of the drag-reduction effect of catfish enabled by mucus. (c) HWTs printed on a silicate glass to collect condensed water droplets and regenerate condensing sites. The blue semi-cylinders represent the HWTs (1 mm in diameter), and the yellow arrows indicate the droplet pumping direction. (d) Schematic diagram of the molecular structure of HWTs. (e) ESEM images of rough HWT surfaces resulting from the drying process. (f) Underwater AFM images of the surface morphologies of dry (left) and wet (right) HWTs.

A promising strategy to circumvent this barrier involves creating the motion trajectory and orienting droplet movement to avoid droplet overgrowth [6,11,12]. Many bionic functional surfaces, including lotus leaves, pitcher plants, cactuses, and spider silks, have inspired micro-/nanostructure designs and wetting gradient surfaces used in different fields like water collection, printing, and drug delivery. They offer great insights into the droplet driving force resulting from the engineered composition and geometry [13–15]. Among them, for droplet navigation, moisture-harvesting lizards, including the Australian thorny devil (*Moloch horridus*) and Texas horned lizard (*Phrynosoma cornutum*), harvest and ingest water in arid areas [16]. Their integuments contain grooved structures comprising scales surrounded by interconnected capillary channels, which could induce a wettability gradient and Laplace pressure difference [17,18]. Therefore, water droplets of various diameters can be harvested and pumped directionally from the scale surface to the capillary channels between the scales (Fig. 1a). With the capillary channels, even a small volume of water can fully disperse over the lizard's integuments, resulting in easier storage and increased availability to its mouth. Moreover, another necessary process after the droplets’ gathering and coalescence is creating a water track that transports water with low drag. In this regard, the skin of most fish (e.g. catfish) is covered with an epidermal mucus layer, which can reduce swimming drag and enhance adaptability to aqueous environments [19]. Fish mucus, a hydrophilic biopolymer, is a lubricant with an ultralow friction coefficient in water (≤5 × 10^−3^) [20]. A shear layer forms between the water and mucus layer, making swimming smoother and the fish more difficult to catch (Fig. 1b) [19,21]. Thus, the merits of both these species can be integrated to design hydrophilic patterns with engineered geometries and properties for sustainable water harvesting.

Using principles derived from moisture-harvesting lizards and surface-lubricated catfish, we herein report on a new design concept for water harvesting. Our engineered pattern consists of ordered hydrogel fibers printed on silicate glass with an arch structure (Fig. 1c). Along the radial direction of the fiber, the arch structure and the superhydrophilicity of the hydrogel surface synergistically create a difference in Laplace pressure and a surface energy gradient across the hydrogel–glass interface, respectively. Mimicking the droplet spreading selectively into the lizards’ capillary channels, condensed water droplets on silicate glass were pumped to the hydrogel fiber surface along its radial direction, and the condensing sites of the glass were regenerated (Fig. 1a and c). Meanwhile, along the axis, the surface of the hydrogel was partially polymerized with abundant branched dangling chains, forming a precursor water film. As shown in Fig. 1b and d, this simulates the mucus of the catfish, as the superhydrophilic hydrogel fiber surface strongly bonds inner-layer water molecules and forms a shear layer for the outer-layer water molecules to swiftly slide off the condensing surface by gravity with low drag resistance. Results show that the engineered hydrogel fibers functioned as tracks for water transport, synchronously enabling favorable radial directional pumping and axial drag-reduced sliding of water droplets. The hydrogel water tracks (HWTs) could effectively regenerate condensing sites and achieve sustainable condensation.

## RESULTS AND DISCUSSION

### Characteristics of HWTs

HWT was fabricated by interpenetrating sodium alginate (SA) and polyvinyl alcohol (PVA) polymer chains. The HWT was designed with an arch structure and was aligned to form anisotropic patterns (Fig. 1c and d). According to environmental scanning electron microscopy (ESEM), the HWT surface morphology varied significantly during dehydration. Thorough dehydration resulted in dramatic shrinkage (Fig. S1), with ridges and trenches forming along the extensively dried HWTs (Fig. 1e). The HWT surface morphology was also studied *in situ* using underwater atomic force microscopy (AFM). The dry HWT surface was rough (root-mean-square roughness, *R*_q _= 16.9 nm) at the nanoscale with numerous wrinkles (Fig. 1f, Fig. S2). After soaking in water, the surface wrinkles of HWT disappeared, with the root-mean-square roughness decreasing by 92.5% (*R*_q _= 1.26 nm). This demonstrated that water molecules could interact with the polymeric chains of HWTs, supporting the skeleton and the formed arch structure.

FTIR spectroscopy was utilized to further verify the interactions between water molecules and HWT. The HWT comprised an interpenetrating polymeric network of SA–PVA and strongly bonded water molecules (Fig. S3a). The broad peak centered at 3294 cm^−1^ (∼3645 to 2700 cm^−1^) was attributed to –OH stretching on the HWT surface, which endows the HWTs with superhydrophilicity to induce the directional droplet movement similar to the moisture-harvesting lizard. When swollen in water, this broad peak was strengthened (Fig. S3b). No absorption peaks were observed at 3756 and 3656 cm^−1^, and the H–O–H bending vibration (∼1630 cm^−1^) overlapped the characteristic –COOH peaks, suggesting that most water molecules on the hydrogel surface were not free [22,23]. The characteristic methyl and methylene peaks around 2920 cm^−1^ weakened significantly after hydration, indicating partial polymerization of the HWT surface because of its polymerization process against hydrophobic air [24–26]. Owing to the branched dangling chains, the synthesized HWT exhibited great hydration ability, and the surface polymer chain concentration decreased significantly in the presence of water molecules, forming a well-developed hydration layer to mimic catfish mucus (Fig. 1d).

### Directional pumping across the glass–HWT interface

**Figure 2.**
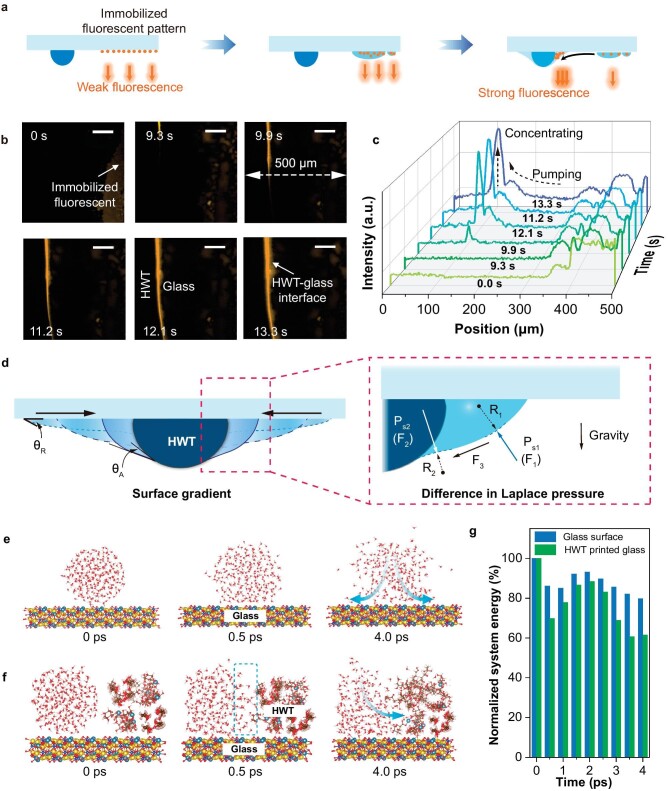
The directional pumping effect of water molecules at the HWT–glass interface. (a) Schematic illustration of the method used to detect the pumping effect of HWTs. Pre-dipped fluorescent molecules enable the tracing of water molecule movement and increase detection sensitivity. The orange straight arrows represent the fluorescence. (b) Fluorescence signals before and after condensation illustrate the directional pumping of water molecules from the glass surface to the HWT (scale bar: 100 μm). (c) Average fluorescence intensity vs. position at various times after condensation. (d) Surface energy gradient and difference in Laplace pressure (inset) between the glass and the HWT. The black solid arrows indicate the direction of the induced pumping effect. MD simulations of water molecule motion over (e) a silicate glass control and (f) an HWT-printed glass surface. (g) The decrease of system energy during motion of the water molecules.

We designed HWTs with arch structures and utilized fluorescence to visualize the motion of condensed water droplets since the HWT pumping effect is the first step for sustainable condensation. First, HWT was placed upside down, in its condensing condition, and was observed by an inverted microscope from the bottom. A fluorescent pattern was immobilized on the glass 200 μm away from an HWT (Fig. 2a). Fluorescent molecules were redispersed in the condensed droplets to show the droplet motion trajectory. Following condensation for 9.3 s, condensed droplets grew gradually to contact the HWT–glass interface, and a strong fluorescence signal appeared at the HWT–glass interface, confirming directional water pumping from the glass to the HWT. An incline of 0.5° allowed rapid water transfer along this interface (Fig. 2b), with water penetrating the entire microscope field in 0.9 s. Moreover, the fluorescence image showed that water was mainly confined within ∼50 μm of the HWT (Fig. 2c). Notably, the fluorescence intensity was nearly 3-fold compared to the immobilized fluorescence pattern, which showed a concomitant decrease. This indicates that water droplets were directionally transferred from the glass toward the HWT, confirming the pumping effect.

Similar to the droplet pumping effect of moisture-harvesting lizards, droplet movement on the HWT–glass interface can be ascribed to differences in affinity for water molecules between the HWT and silicate glass. The water contact angle on the dry HWT film was 35.7°, much lower than that on bare glass (52.9°; Fig. S4a and b), illustrating its better affinity for water molecules. This gap widened further in wet HWT. The HWT surface was rich in oxygen-containing functional groups, which could form hydrogen bonds to hold water molecules in the polymeric network. The retained water molecules formed a precursor water film on the HWTs, leading to the rapid spread (instantaneous contact angle ∼16.7°) of subsequent water molecules. As a result, the HWT surface became super-wettable (Fig. S4c). This difference in hydrophilicity induced a chemical wetting gradient, as given by Eq. 1 [27,28]:


(1) \begin{equation*}
{{F }} = \mathop \int \nolimits_{{{\mathrm{L}}}_{\mathrm{g}}}^{{{\mathrm{L}}}_{\mathrm{h}}} {\mathrm{\gamma }}\!\left( {{\mathrm{cos}}{{\mathrm{\theta }}}_{{A}} - {\mathrm{ cos}}{{\mathrm{\theta }}}_{{R}}} \right){{\ dl}},\end{equation*}

where ${\theta }_A$ and ${\theta }_R$ are the advancing and receding angles of a water droplet at the glass–hydrogel interface, respectively, $\gamma $ is the surface tension of water (73 mN m^−1^), and *dl* is the integration variable along the length from the glass to the HWT. Compared to ${\theta }_R$, ${\theta }_A$ was negligible, driving the water droplets from the less hydrophilic glass to the more hydrophilic HWT (Fig. 2d). Ideally, ${\theta }_R$ and ${\theta }_A$ are 52.9° and 16.7°, *l* is the horizontally projected length of the 1/2 HWT (∼500 μm), so the wetting gradient *F* is estimated as ∼0.013 mN.

The arch geometry of HWT is also critical for the pumping effect, which acts like the meniscus-mediated coarsening effect [29]. It facilitates generating a difference in the Laplace pressure (Young–Laplace equation) [29,30]:


(2) \begin{equation*}
{P}_{\mathrm{s}} = {\mathrm{ \gamma}}\! \left(\frac{{\mathrm{1}}}{{{R}_{\mathrm{2}}}}{\mathrm{ + }}\frac{{\mathrm{1}}}{{{R}_{\mathrm{1}}}} \right),\end{equation*}

where *R*_1_ and *R*_2_ are the radii of the water surface curvature. Ideally, *R*_1_ and *R*_2_ are ∼1.63 mm and ∼ −0.77 mm (Figs S4b and S5c), *P*_s_ is estimated as ∼ −50 Pa, which points to the exterior of the droplet. For its direction, as illustrated in Fig. 2d (inset), the contact angle of water on bare glass creates a downward curvature with radius *R*_1_, inducing *F*_1_ pointing to the droplet's interior. When the droplet completely deposits on the glass, the droplet is horizontally symmetrical, so no net force exists horizontally. When the droplet reaches HWT, the superhydrophilicity of HWT induces a tiny contact angle at the water–hydrogel surface, creating an upward curvature with radius *R*_2_ and *F*_2_ pointing to the exterior of the droplet (Fig. S5). The resultant force (*F*_3_) of *F*_1_, *F*_2_, and gravity points toward the HWT, driving the water droplet to move from the glass to the HWT. Overall, the surface energy gradient and difference in Laplace pressure cooperatively induce the HWT pumping effect.

These findings were further verified by using molecular dynamic (MD) simulations. A water droplet released on bare glass spread rapidly, with the boundary becoming unclear after 4.0 ps owing to the affinity of water for the glass surface and Brownian motion (Fig. 2e). However, droplet boundary deformation occurred axisymmetrically with no directional tendency. Conversely, the tendency was evident in the HWTs (Fig. 2f). The water molecules moved quickly, encountering the HWT within 0.5 ps. Subsequently, the top edge of the water droplet tilted toward the HWT, and water molecules even entered the HWT skeleton after 4.0 ps. The free spread of water droplets on bare glass is an entropy-increasing but energy-decreasing process (by ∼20%) as water molecules move spontaneously to areas with higher surface energy (Fig. 2g). Comparatively, the system energy decreases by ∼40% for water molecules spreading near HWTs, demonstrating that HWTs provide additional forces that account for the directional pumping effect. Therefore, the semicircular convex shape and superhydrophilicity of the HWTs are critical for directional droplet pumping, mimicking the capillary channels of lizards to directionally pump droplets and regenerate condensing sites.

### Drag-reduced water sliding along HWTs

After pumping droplets into the HWT, rapid movement along the HWT is necessary to ensure sustainable pumping and avoid surface flooding [31]. As shown in Fig. S6, once the droplet contacts the HWT surface, an inclination of only 0.5° can lead to a quick spreading and rapid water sliding within 0.1 s and 1.0 s, respectively. The instantaneous advancing angle at 0.1 s was as low as 2.7° and quickly disappeared. In contrast, the droplet tended to pin onto the glass surface (Fig. S7). When inclined at 90.0°, the advancing angle increased from 55.4° to 74.7°, but the droplet was still unable to roll off the glass surface. Therefore, it was easier to remove water on the HWT surface than on glass.

**Figure 3.**
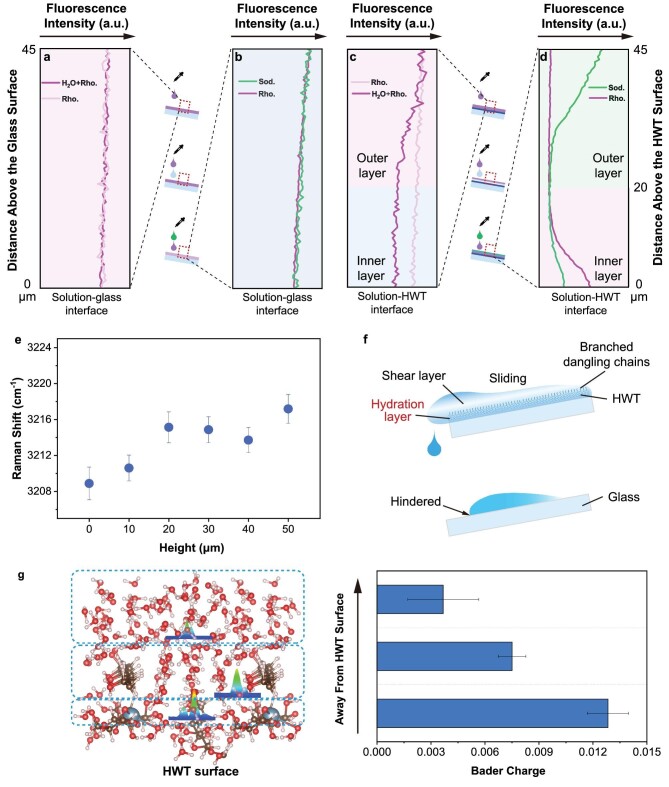
Mechanism of water transport on the HWT surface. (a) A rhodamine B droplet sliding on the glass fully mixing with former droplets. (b) Successive rhodamine B and sodium fluorescein droplets sliding and fully mixing on the glass surface. (c) A rhodamine B droplet sliding on the hydrogel surface illustrates hydration layer formation, with subsequent water droplets advancing without direct contact with the hydrogel surface. (d) Successive rhodamine B and sodium fluorescein droplet sliding illustrate the water division on the hydrogel surface into inner and outer layers. The background shading represents the color of the droplet, where Sod. corresponds to green, Rho. corresponds to pink, and water corresponds to blue. (e) Change in Raman shift at ∼3220 cm^−1^ upon water molecules approaching the hydrogel surface. (f) Schematics of the hindered droplet transport on bare glass control and rapid sliding on HWT-printed glass. (g) DFT-calculated charge transfer intensity for water molecules at various distances from the HWT interface.

Ionic fluorescent dyes rhodamine B and sodium fluorescein were used to monitor the movement of surface water molecules. As shown in Fig. 3a, sliding a rhodamine B droplet along the glass surface formed a trail with a uniform fluorescence signal profile 45 μm above the surface. Similarly, after wetting the bare glass surface, the subsequent rhodamine B mixed thoroughly with water in the previously formed thin film, resulting in a fluorescence signal resembling that of the rhodamine B droplet only (Fig. 3a). Furthermore, when the rhodamine B droplet was followed by a sodium fluorescein droplet, mixing resulted in a uniform vertical distribution of these two fluorescences on the glass surface (Fig. 3b).

Water molecule movement on the hydrogel surface differed. With only the rhodamine B droplet sliding on the hydrogel surface, the vertical distribution was uniform, similar to that on the glass surface (Fig. 3a and c). However, when the hydrogel surface was first wetted by water, the subsequently sliding rhodamine B mainly distributed in the outer layer of the water film, indicating that it advanced without approaching the hydrogel surface, remaining distributed in the outer layer of the water film (Fig. 3c). This phenomenon was further verified by successively sliding rhodamine B and sodium fluorescein on the HWT surface (Fig. 3d). Sodium fluorescein completely washed away the outer rhodamine B layer, whereas the inner rhodamine B layer was still preserved. Thus, sodium fluorescein and rhodamine B mainly occupied the outer and inner layers, respectively. These results indicate that the inner-layer water molecules are more inert and have a stronger affinity for the hydrogel surface than those in the outer layer.

MicroRaman was applied to quickly switch the focus between the glass and the HWT surface to monitor the O–H stretching modes of water molecules at different heights away from these two surfaces. The obtained signals were differentiated using Gaussian functions (Fig. S8). The peaks at ∼3220 cm^−1^ (Peak A) and ∼3410 cm^−1^ (Peak B) corresponded to the in-phase and out-of-phase vibrations of water molecules with four hydrogen bonds, respectively, whereas that at ∼3610 cm^−1^ (Peak C) corresponded to the O–H stretching vibrations of weakly or non–hydrogen-bonded water molecules [32,33]. Upon approaching the hydrogel surface, the percentage of weakly bonded water molecules decreased, indicating an increase in water molecules with tetragonal structures (Fig. S9). A corresponding frequency decrease in Peak A suggested O–H bond elongation due to the hydrogen bonds between the interfacial water molecules and the –OH and –COOH groups of the hydrogels being stronger than those between bulk water molecules (Fig. 3e) [34]. The superhydrophilic branched dangling chains on the HWT surface held water molecules even after the water droplets left the surface, thus improving the precursor water film around the condensing droplets and facilitating the rapid formation of a stable water film (Fig. S10) [35,36]. Comparing the droplet movement on both wet and dry HWTs, we found that on the dry HWT surface, successive droplets were pinned and accumulated into a larger one (Fig. S11a, movie S1), similar to the bare glass surface (Fig. S11c). Comparatively, successive droplets released on the wet HWT surface could rapidly slide away without flooding the surface (Fig. S11b and movie S2). Therefore, the formation of precursor water film is crucial to act as a lubricant layer between the inner and outer water layers to make the outer water molecules advance without direct hydrogel contact, thus dramatically reducing drag (Fig. 3f). However, on silicate glass, water molecules advanced as a whole with successive sliding and friction against the glass surface.

Hydration layer formation relies strongly on hydrogen bonds, which are electrostatic interactions or covalent chemical bonds that are closely related to electron distribution [37,38]. Using density functional theory (DFT) calculations, the charge transfer intensity was determined to evaluate interactions between a water molecule and its neighbors. Away from the HWT surface, charge transfer between water molecules was weak, corresponding to natural hydrogen bonding in liquid water (Fig. 3g). In bulk water, hydrogen bond formation between neighboring molecules is statistically homogeneous and isotropic, leading to negligible net charge transfer [39,40]. Upon approaching the HWT surface, the charge transfer intensity increased significantly, eventually becoming 249% greater than that in bulk water, suggesting a loss in symmetry for the interfacial water molecules. Thus, the hydrogen bonds between water molecules and the HWT surface were stronger than those between neighboring molecules in bulk water, leading to a net charge transfer, in agreement with the Raman results [39,41]. However, when water molecules entered the HWT skeleton, the charge transfer intensity decreased dramatically, becoming similar to that away from the HWT surface (Fig. S12). As the water molecules within the HWT skeleton could uniformly form hydrogen bonds with the surface, they remained centrosymmetric, and no net charge transfer was observed. Therefore, the superhydrophilicity of the branched dangling chains on the HWT surface favors hydration layer formation. Similar to catfish mucus, this shear layer enables outer-layer water to slide swiftly on the smooth HWT surface with reduced drag.

### Sustainable water harvesting via directional pumping and drag-reduced sliding

**Figure 4.**
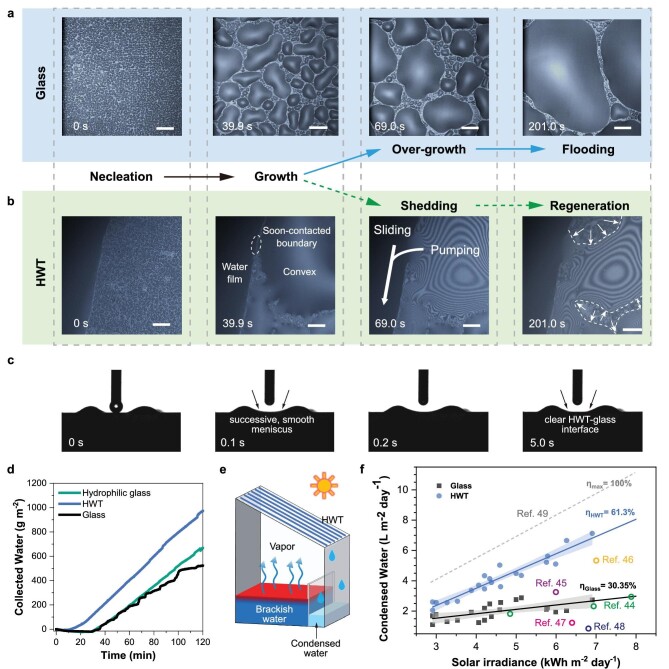
Coupled water droplet growth and directional pumping for accelerated water vapor condensation. (a) *In situ* optical images of rapid water droplet formation, growth, cohesion, and film-forming processes on bare glass control. (b) *In situ* optical images showing rapid condensation, water droplet growth, directional pumping, drag-reduced sliding effect, and condensing site regeneration on HWT-printed glass (scale bar: 100 μm). (c) Optical microscope images of the HWT pumping effect (needle diameter: 0.5 mm). (d) Water droplet collection rates on the HWT pattern and bare glass. (e) Illustration of the pilot study of condensed water collection. (f) Condensed water from outdoor solar evaporation devices with glass and HWT-printed glass condensing cover. The reference data were obtained or calculated from previous reports.

Owing to the unique pattern and properties of the HWT, the condensation behavior observed *in situ* on silicate glass and the HWT pattern differed. HWTs are 1.0 mm hydrogel fibers, and HWT-*x* denotes the interval between HWTs are *x* mm. On silicate surface control, water droplets formed quickly as condensation began (Fig. 4a). Subsequently, the border of these water droplets expanded, resulting in successive collisions and cohesion (diameter >100 μm). Finally, a water film flooded the glass surface, blocking the condensation sites and hindering further condensation (Fig. S13a). Conversely, on the HWT pattern, water droplets formed quickly on the glass (Fig. 4b; 0 s), and a thin water film formed instantly on the HWT (Fig. S14). When the water droplet grew, its boundary expanded and encountered the HWT (Fig. S13; 40.2 s). The water droplet was rapidly pumped by the HWT and transported axially along the HWT, as traced by the small-particle trajectory from the middle of the water droplet to the HWT (Fig. S13b, position 1–4). Finally, the water droplet boundary on the glass receded, condensation sites were regenerated, and new water droplets formed (Fig. 4b; 201.0 s).

The radial pumping and axial sliding of water droplets were verified by placing the HWT-printed surface upside down and using a time-resolved camera from the side. Three representative initial locations were selected to observe the directional pumping effect (Fig. S15). When the water droplet was released directly on the HWT, it spread rapidly (Fig. S15a). Interestingly, this droplet spilled over the HWT boundary before being swiftly dragged back (within 1 s), forming an afterimage. This behavior was because of the balance between the effect of gravity on the water droplet and the HWT pumping effect. Owing to the 0.5° inclination along the HWT, the water droplet left the focal plane within 5 s, regenerating the initial boundary between the HWT and glass. This HWT pumping effect was also observed when water droplets were released at the HWT–glass interface. Even when the water droplet was released between two HWTs, it was quickly pumped to the HWTs and formed a concave meniscus between the HWTs within 1 s (Fig. 4c; labeled with arrows). The meniscus gradually disappeared after the water slid along the HWTs (Fig. S15c, 20 s).

The water collection properties were investigated using HWT-patterned glass as the inclined top of a container. In the container, the relative humidity and temperature were controlled at 97.5 ${\mathrm{ \pm }}$ 0.06% and 40 ${\mathrm{ \pm }}$ 0.2°C, respectively (Fig. S16). For bare glass control, condensation resulted in instant and randomly formed fine water droplets that firmly adhered to the surface, causing fog (Fig. S17), deteriorating the light transmittance by 18.80 ${\mathrm{ \pm }}$ 1% (Fig. S18). Moreover, this adhesion led to minimal harvesting of the water and uneven increases in the mass of collected water, with sharp changes at 67 and 96 min corresponding to the formation of water droplets that were sufficiently heavy to be shed (Fig. 4d). Additionally, the condensation rate on bare glass deteriorated over time owing to the retention of condensed water on the condensation sites. Comparatively, increasing the hydrophilicity of glass can lead to smoother droplet shedding and increase the water collection rate by 27.6% while still suffering from unsustainable condensing sites [2,42]. When the HWT pattern was applied, the formed droplets could be quickly pumped from the glass to the HWTs, and the condensed water was mainly concentrated at the glass–HWT interface with several growing droplets still on the interval between HWTs. No notable flooding happened between the two HWTs (Fig. S17). The condensed water was first collected after 10 min, earlier than on bare glass and hydrophilic glass control. Moreover, the condensation rate increased by 85.9% and remained stable, demonstrating reduced retention and sustainable condensation of water droplets on the HWT pattern. After condensation, the light transmittance of HWT-printed glass only decreased by 4.030 ± 2%, indicating the quick shedding of the droplets (Fig. S18). As shown in Fig. S19, when the HWTs were closer (HWT–0.4; 0.4 denotes the gap (mm) between HWTs), condensed water droplets could be collected earlier than HWT–1.0, attributable to the decreased pumping distance ensuring earlier shedding of the condensed droplets on the glass. However, when intensive condensation occurred, droplets grew rapidly to contact the left and right HWT concurrently, resulting in partial flooding between the neighboring HWTs and slightly deteriorating condensation performance. In contrast, when the interval between the HWTs (HWT–2.0) was enlarged, water collection was delayed compared to HWT–0.4 and HWT–1.0 since the droplets needed to grow larger to contact the HWT and be pumped away. As expected, the delayed shedding of the droplet gradually affected condensing performance. Therefore, directional pumping of HWT is critical for sustainable condensation, and its most effective pumping range was around 200–500 μm.

Solar evaporation has been widely regarded as the zero-carbon water purification technique and has received intense attention for its potential to resolve the clean water crisis. However, previous solar evaporation studies mainly focused on solar-to-vapor conversion, but the vapor-to-water process has long been overlooked. Condensation has been regarded as the bottleneck for acquiring purified water in sunlight-generated water vapor [43–45]. Thus, we applied the HWT-printed glass to solar evaporation devices under natural conditions to further demonstrate its applicability and scalability. The devices with a larger condensing area (200.0 × 200.0 mm^2^) were placed outside to collect purified water under natural daily irradiance and other weather conditions. We applied our HWT-printed glass as the condensing cover of solar evaporation equipment and used bare glass as the control group (Fig. 4e). For the bare glass control, the water production rate was within 1.12 to 2.88 L m^−2^ day^−1^, corresponding to an average solar-to-water efficiency of 30.34%, comparable to previous reports (Fig. 4f, Table S1) [44–48]. In contrast, with the HWT pattern, the water production rate drastically increased, ranging from 2.05 to 7.12 L m^−2^ day^−1^ (efficiency ≈61.3%), close to the theoretical maximum [49]. Evidently, with an enlarged surface area, the distance for the droplets to be shed off also increases, further challenging the condensing performance of bare glass. As expected, the elevation for larger-sized HWT-printed glass is 109%, higher than that of the lab scale (85.9%), demonstrating that the pumping effect enabled by HWT patterns promotes condensation by accelerating the droplets away from the glass edges to be shed and is especially critical in large-area sustainable condensation. Moreover, HWT is stable in natural environments (solar irradiance), the release of Ca^2+^ is negligible, and the produced water with the HWT (∼10 μS cm^−1^) showed no difference from that of bare glass (Figs S20 and S21). This demonstrated that HWT could be scaled up to increase the condensing rate without external energy input and with no threat to the quality of the condensed water.

## CONCLUSION

In summary, we successfully incorporated nature-inspired principles into an artificial system for designing the geometry and surface properties of hydrogel fibers. The anisotropic hydrogel patterns with arch structures printed on silicate glass showed rapid and sustainable condensation for atmospheric water harvesting. By synergistically combining radial and axial droplet manipulation, randomly dispersed water droplets were directionally pumped to the HWT surface and slid swiftly with negligible drag. Consequently, the condensing sites were regenerated, facilitating effective and sustainable condensation. This strategy fully utilized the classical merits of hydrogels and is also feasible on typical heat-conducting materials such as copper and aluminum (Fig. S22), making it more flexible and applicable for water purification, extraction, and energy conversion techniques related to condensation processes, including water desalination, solar water production, fog harvesting, and heating/cooling systems.

## METHODS

### Measuring the water collection properties

The HWT-printed glass (90 × 90 mm^2^) served as the condensation plate with an inclination of 19°. Meanwhile, the condensing surface temperature was 20°C. In the container, 100 mL of water was added and heated to 70°C for evaporation. The relative humidity and temperature inside the container were both recorded by a computer (UNO, Arduino). After the water vapor had condensed on the HWT-printed glass, the condensate was collected by a water container, and its weight was monitored by an analytical balance (ARR224CN, OHAUS Adventurer) connected to a computer. The pilot solar evaporation study was carried out under natural irradiance. Two acrylic containers were used as water tanks and were well sealed with two top glass covers (20 × 20 cm^2^) to avoid the escape of water vapor. Thus, the water vapor could be condensed on the top cover and driven by gravity for collection. The water was simulated brackish water (NaCl (1.0006 g L^−1^), CaCl_2_ (0.2775 g L^−1^), Na_2_SO_4_ (0.4925 g L^−1^), MgCl_2_·6H_2_O (0.4177 g L^−1^)) and the water surface was covered by a layer of solar evaporator reported previously to utilize sunlight for accelerated brackish water desalination [50]. The only difference between the two pieces of equipment was that one top cover was silicate glass (control) and the other was HWT-printed glass.

### Characterization of the water droplet movement

The contact angle analyzer (Dataphysics OCA15EC, Germany) was focused on the edge of the glass plate and the middle of two HWTs printed on its surface. The glass plate was inclined to 0.5° along the direction of the HWTs. The syringe needle was initially located at the middle of two HWTs, the edge, and the top of one HWT. The water droplet was carefully dripped onto the surface, and water droplet movement was monitored at 10 fps by a digital camera. The optical and fluorescence images during condensation were recorded by a digital camera and inverted laser confocal fluorescent microscope (IX83ZDC, OLYMPUS). A printed glass, inclined at 0.5° along the direction of the HWTs, was used at the microscope stage. A bare glass plate was set as a reference. Water vapor was generated by an ultrasonic nebulizer and directed to the observation field. Fluorescein sodium and rhodamine B were dissolved to give 0.1 wt% aqueous solutions, serving as fluorescent dyes. Fluorescein sodium was excited at 488 nm and measured at 525 ± 22.5 nm, whereas rhodamine B was excited at 561 nm and measured at 607 ± 18 nm. In the water droplet movement test during condensation, a tiny drop of rhodamine B was pre-dripped onto the glass plate between two HWTs and air-dried. Then condensation was initiated, and the rhodamine B signal was monitored. In the water droplet movement test on the surface of hydrogel and bare glass, water, rhodamine B, and fluorescein sodium droplets were dripped onto the hydrogel in different sequences. The signals of both rhodamine B and fluorescein sodium were separately recorded against the distance above the hydrogel surface to depict the movement behavior of the water on the hydrogel surface.

## Supplementary Material

nwad334_Supplemental_Files
