# Supplementary material for: Pumping and sliding of droplets steered by a hydrogel pattern for atmospheric water harvesting
Source: Natl Sci Rev. 2023 Dec 28;10(12):nwad334. doi: 10.1093/nsr/nwad334 (PMC10829482; doi:10.1093/nsr/nwad334)
Supplement: nwad334_Supplemental_Files [file nwad334_supplemental_files.zip › Supplementary_materials-for_publication.docx]

Supplementary Information

*Wei Zhang, Qinghua Ji, Gong Zhang, Haozhi Wang, Zhenao Gu, Chengzhi Hu, Huijuan Liu, Zhiyong Jason Ren, and Jiuhui Qu*

**Table of Contents**

**Supplementary Fig. 1** Environmental scanning electron microscope (ESEM) images of the dried HWT surface.

**Supplementary Fig. 2** (Under-water) Atomic force microscopy (AFM) of the HWT with the roughness labeled.

**Supplementary Fig. 3** FTIR spectrum showing the IPN structure of the HWT constituents.

**Supplementary Fig. 4** Optical photographs showing the behaviors of the droplet on different surfaces.

**Supplementary Fig. 5** The upward curvature over the HWT surface.

**Supplementary Fig. 6** Optical photographs showing the spreading and sliding of the droplet on HWT surface.

**Supplementary Fig. 7** Optical photographs showing the pinning of the droplet on glass surface with an inclination

**Supplementary Fig. 8** Raman spectra of water molecules at different distances from the hydrogel surface.

**Supplementary Fig. 9** The percentage of the free water molecules as a function of the distance from the HWT surface.

**Supplementary Fig. 10** The Raman spectrum of the water molecules on different surfaces before and after water rinsing.

**Supplementary Fig. 11** Comparisons of the water droplet sliding on different surfaces.

**Supplementary Fig. 12** The net charge transfer of the water molecules in the HWT and at different distances from the HWT surface.

**Supplementary Fig. 13** Optical photos of the condensing process on different surfaces.

**Supplementary Fig. 14** Optical microscope photograph of the water film forming process during condensation.

**Supplementary Fig. 15** Optical microscope photograph showing the pumping effect of the HWT.

**Supplementary Fig. 16** Temperature and humidity within the condenser.

**Supplementary Fig. 17** Optical photos of the glass surface with(out) different HWTs on it during the condensation process.

**Supplementary Fig. 18** Light transmittance of the glass surface with(out) different HWTs on it during the condensation process.

**Supplementary Fig. 19** The influence of the intervals between HWTs on the water droplet collection rates.

**Supplementary Fig. 20** Conductivity of the produced water with bare glass and HWT pattern.

**Supplementary Fig. 21** Ca^2+^ concentration of the collected water during outdoor experiment.

**Supplementary Fig. 22** The HWT printed on (a) aluminum and (b) copper foil, respectively.

**Table S1** The collected condensed water of outdoor solar evaporation devices.

**MATERIALS AND METHODS**

**Materials.** Polyethyleneimine (PEI, Mw~1300, 50% in H_2_O) was purchased from Sigma-Aldrich. CaCl_2_, sodium alginate (SA), and polyvinyl alcohol 1750±50 (PVA) were purchased from Sinopharm Chemical Reagent Corporation (China). Rhodamine B and fluorescein sodium were purchased from MACKLIN.

**Preparation of hydrogel water track (HWT) coating.** In a typical preparation procedure, SA was stirred and dissolved in ultrapure water by the mass fraction of 4 wt%, and PVA was stirred at a temperature of 95 ℃, forming a solution of 10 wt%. SA and PVA solutions were mixed by a ratio of 2:1 (v/v) and stirred for 1 h to form a homogeneous solution, which was denoted as the HWT precursor. PEI was diluted to 10 wt% by ultrapure water. A glass plate was subsequently cleaned with acetone, ethanol, and water. Then this glass plate was immersed in the PEI aqueous solution for 30 s and taken out to be dried at room temperature. Syringe nozzles with holes (1.0 mm in diameter) in a row were fabricated by 3D printing, and the spacing between aligned holes was designed at 1.0, 0.4 and 2.0 mm. Before coating the HWT onto the glass plate, the glass plate was frozen to 263.15 K with liquid nitrogen. A syringe pump was used to control the injection rate of the HWT precursor (1.5 mL min^-1^), and the scan rate of the syringe nozzle was set as 5 mm s^-1^. After coating, the precursor quickly got frozen, and the glass plate was stored in the refrigerator at 253.15 K for 2 h. Then it was taken out to recover to nearly 0 ℃ and this freeze-thaw process was repeated 3 times for PVA to form a stable network. Then it was immersed in 0.25 mol L^-1^ CaCl_2_ solution for 30 min and then taken out for repeated rinsing.

**Characterization of the HWTs.** The surface conditions of HWT were obtained by environmental scanning electron microscopy (Quattro C, ThermoFisher). The morphology images and roughness data were obtained in ambient air and underwater by atomic force microscope (FASTSCANBIO, Bruker). The surface functional groups were characterized by Fourier transform infrared spectrometer with attenuated total reflectance accessories (FTIR-ATR, Excalibur 3100, Varian). The vibration modes of water molecules were recorded by MicroRaman spectroscopy (Renishaw, inVia-Qontor).

**Molecular Dynamic (MD) simulation.** The models consisting of bare glass (Na_2_CaSiO_4_) surface and glass with HWT decorated were built, respectively. and the COMPASS forcefield was implemented to optimize the structures of interest [1]. Generally, the thermal vibrations of bulk atoms within the solid were little and can be neglected [2], so all the bottom 2 layers of glass atoms were fixed at their bulk-like positions. The electrostatic interactions were handled by the Ewald summation technique, and the Ewald accuracy was set to 1.0 × 10^-4^ kcal mol^-1^ [3]. Van der Waals interactions were described by the atom-based summation approach. The cutoff distance, spline width, and buffer width were set as 15.5 Å, 1 Å, and 0.5 Å, respectively. The Andersen algorithm was employed to control the temperature of the simulation at different levels [4]. The MD simulation was performed under an NVT ensemble with a time step of 1.0 fs and a total simulation time of 500 ps.

**DFT simulation.** All the calculations were performed within the framework of the density functional theory (DFT) as implemented in the Vienna Ab initio Software Package (VASP 5.4.4) code within the Perdew–Burke–Ernzerhof (PBE) generalized gradient approximation and the projected augmented wave (PAW) method [5-8]. The cutoff energy for the plane-wave basis set was set to 450 eV. The ultrasoft pseudo-potential was employed to describe the interaction between valence electrons and the ionic core. Monkhorst–Pack special k-point meshes of 1 × 1 × 1 were proposed to carry out geometry optimization and electronic structure calculation. During the geometry optimization, all atoms were allowed to relax without any constraints until the convergence thresholds of maximum force and energy were smaller than 0.01 eV/Å and 1.0 × 10^−5^ eV/atom, respectively. A vacuum layer of 12 Å was introduced to avoid interactions between periodic images.


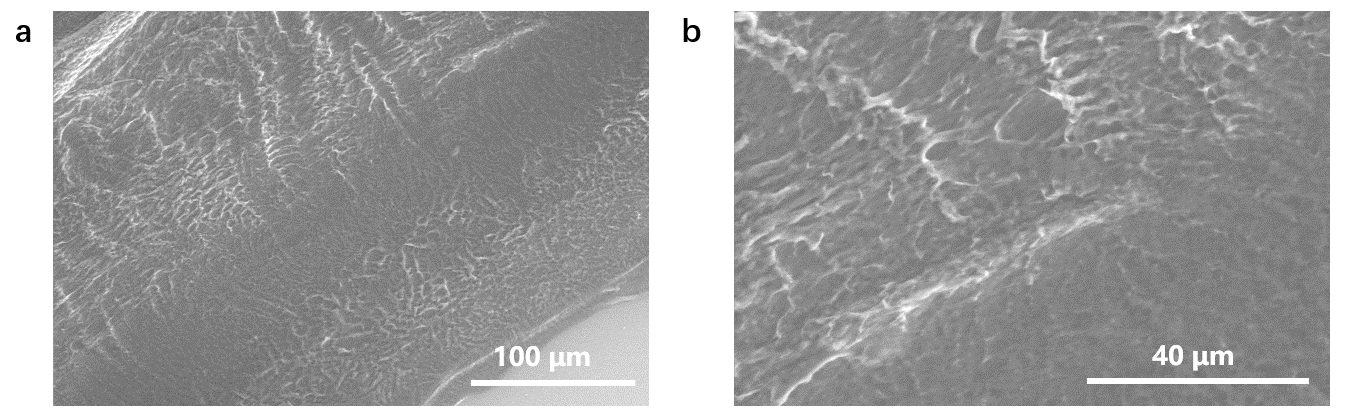


**Supplementary Fig. 1** Environmental scanning electron microscope (ESEM) images of the dried HWT surface. (a) Distant view. (b) Enlarged view. Ridges and trenches were all over the surface of the HWT.


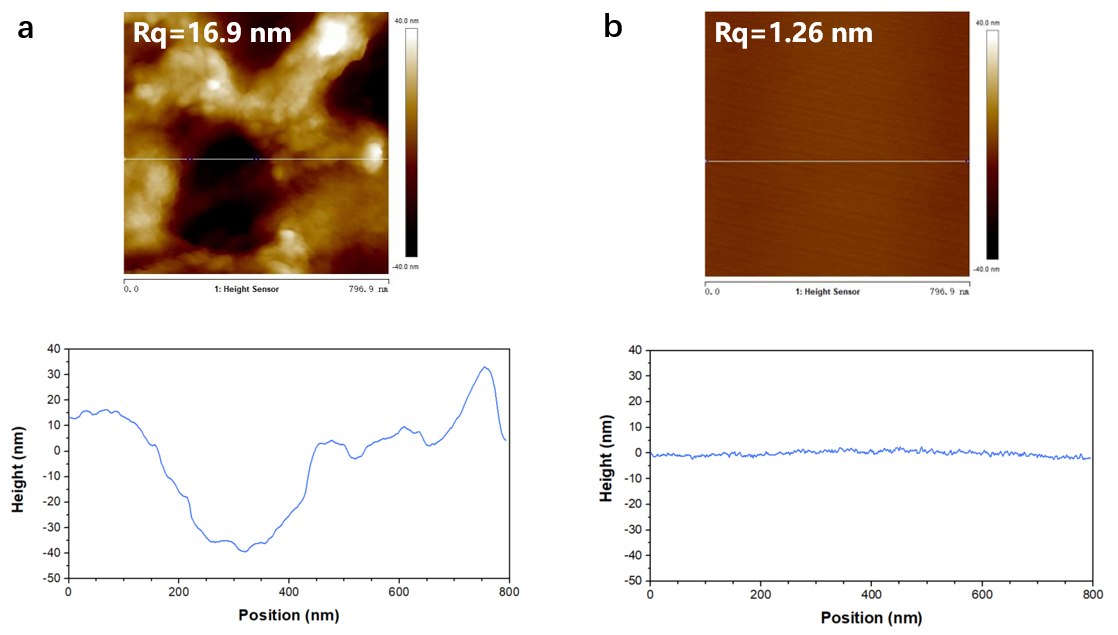


**Supplementary Fig. 2** (Under-water) Atomic force microscopy (AFM) of the HWT with the roughness labeled. (a) The upper image showed the top view of the HWT in the dry state. The lower image showed the profile of the line in the upper image. (b) The upper image showed the top view of the HWT underwater. The lower image showed the profile of the line in the upper image.

Under the dry state, peaks were observed at 1589 and 1411 cm^-1^, corresponding to –COOH antisymmetric and symmetric stretching in the SA molecular skeleton[9,10], and at 1093 and 1327 cm^-1^, attributed to C–O stretching and C–H deformation in PVA[11,12].


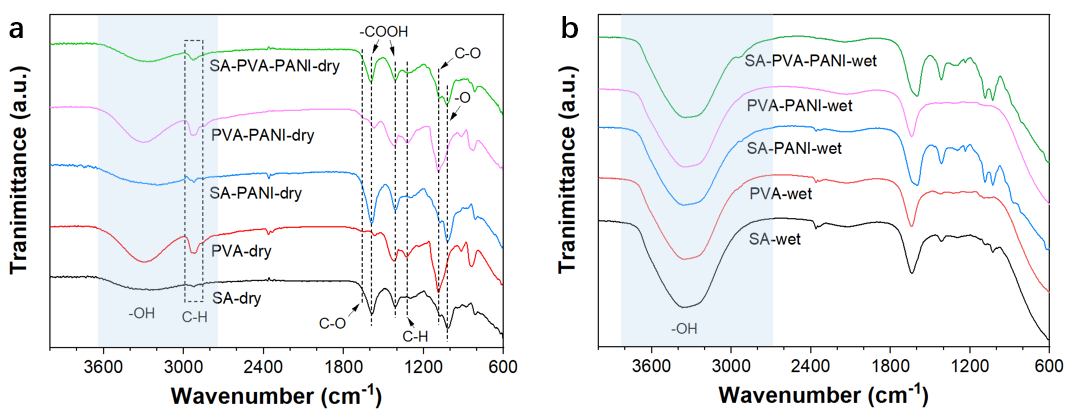


**Supplementary Fig. 3** FTIR spectrum showing the IPN structure of the HWT constituents. (a) In the dry state, oxygen-containing groups were found on SA and PVA surfaces. (b) In the wet state, lots of water molecules were absorbed in the skeleton of HWT, showing a broad peak from ~3645-2500 cm^-1^.


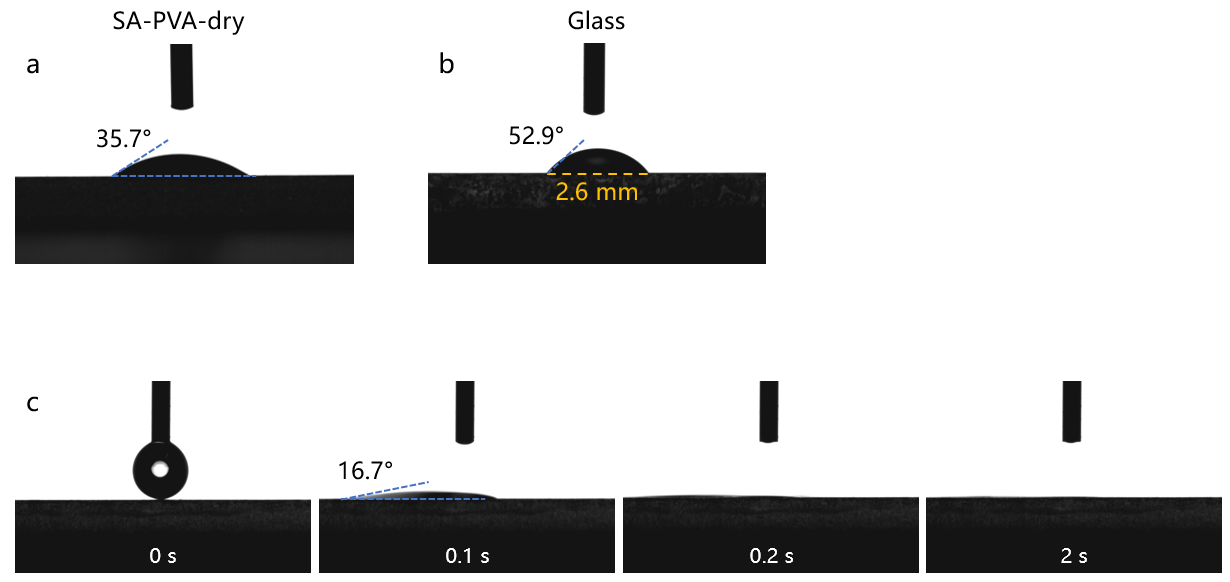


**Supplementary Fig. 4** Optical photographs showing the behaviors of the droplet on different surfaces. (a) The contact angle on the dry HWT surface. (b) The contact angle on the bare glass. (c) The rapid spreading of the water droplet on the wetted HWT surface.


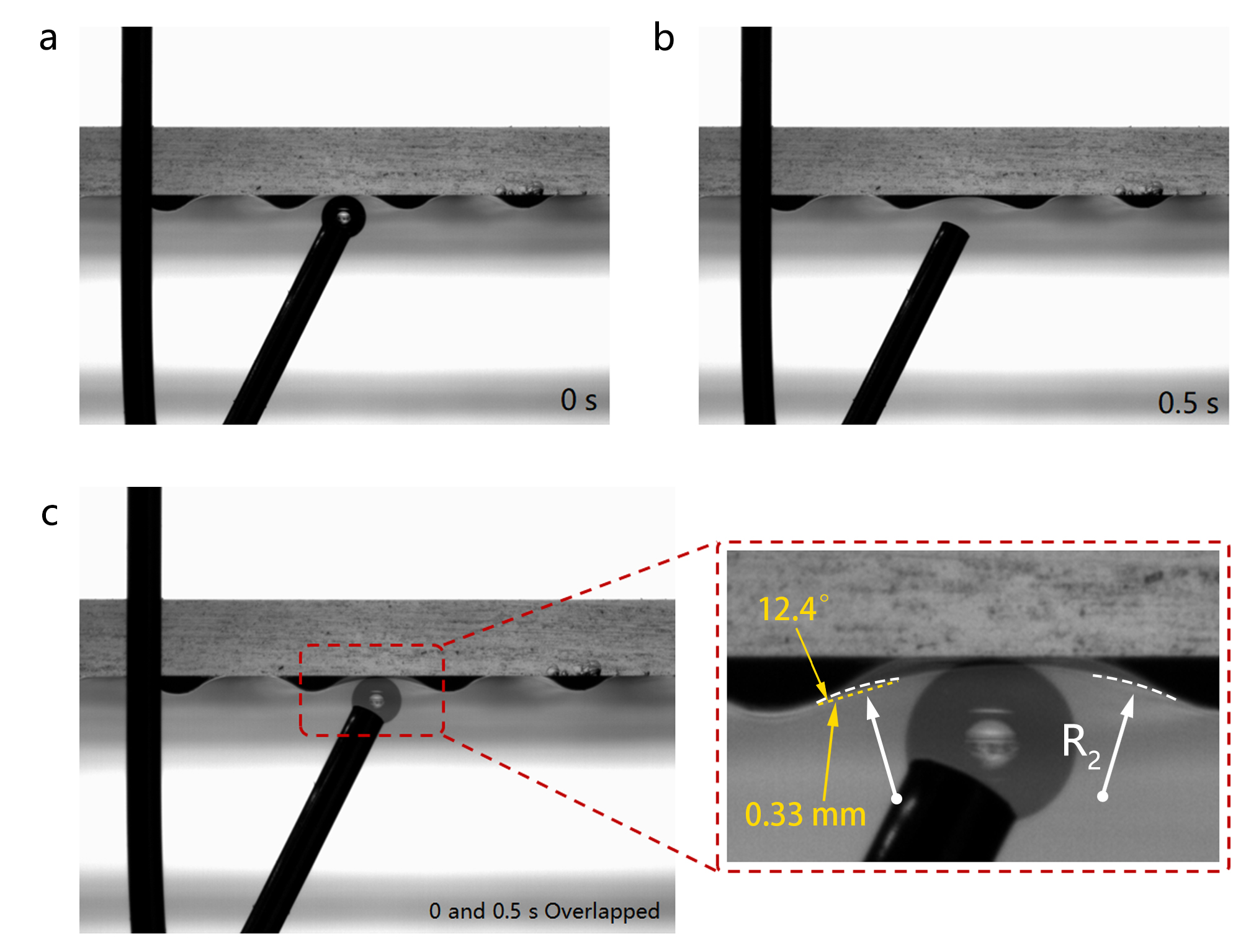


**Supplementary Fig. 5** The upward curvature over the HWT surface.


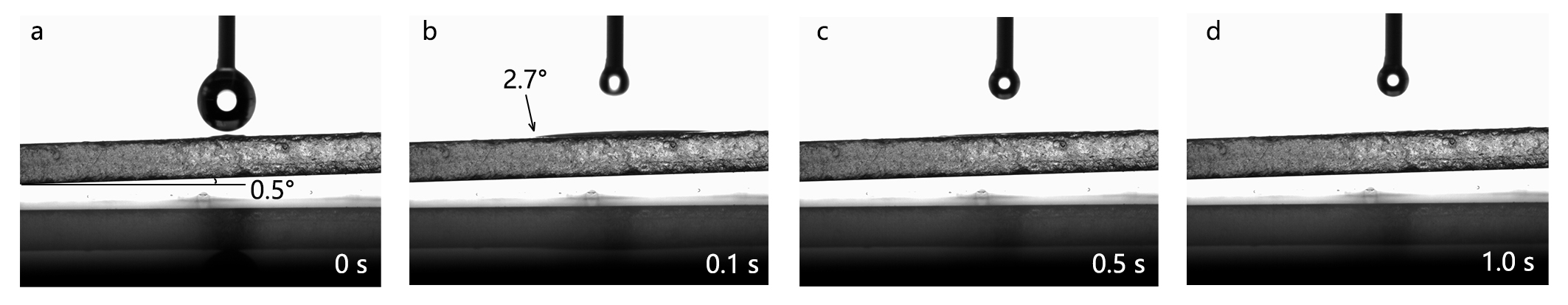


**Supplementary Fig. 6** Optical photographs showing the spreading and sliding of the droplet on HWT surface.


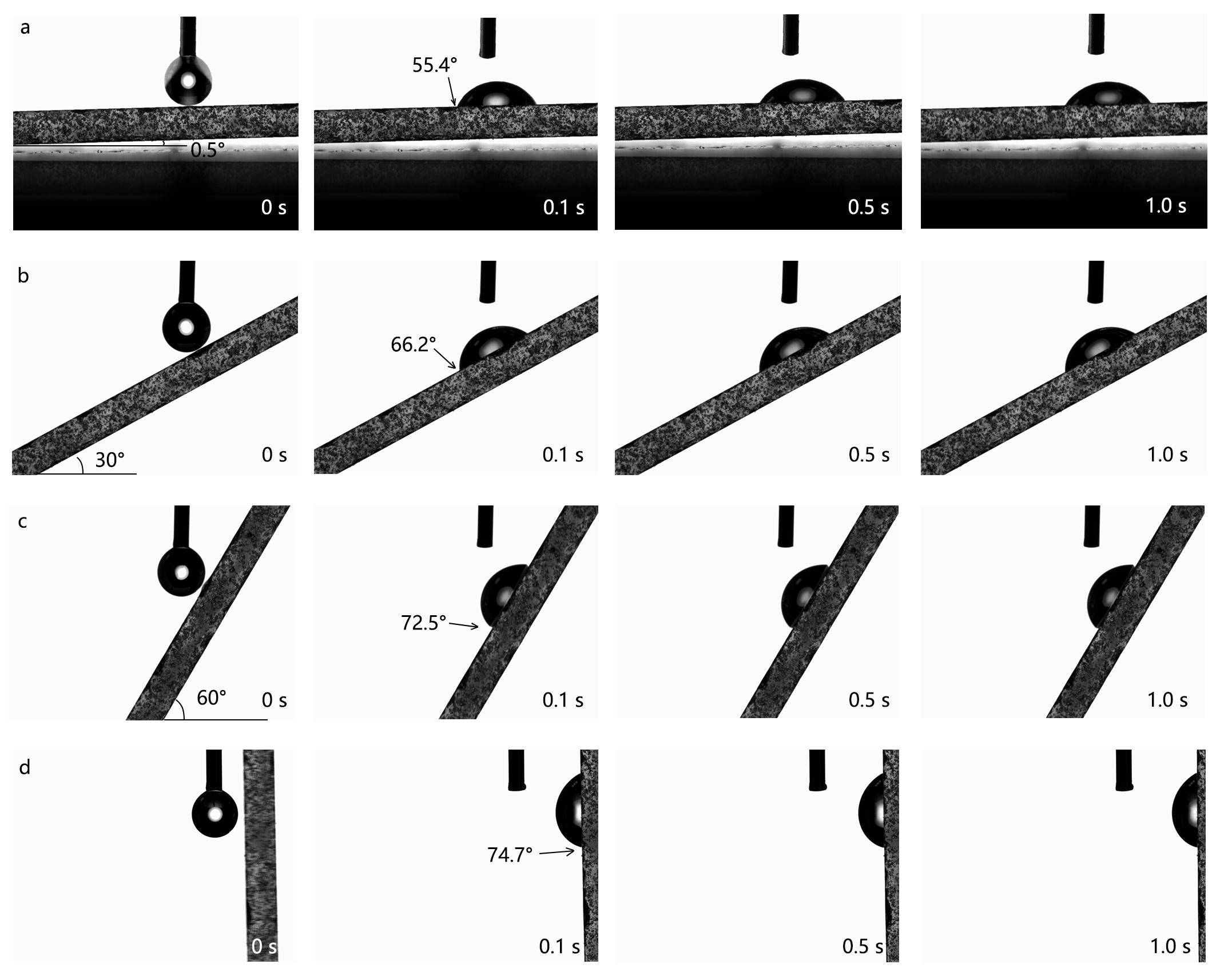


**Supplementary Fig. 7** Optical photographs showing the pinning of the droplet on glass surface with an inclination of (a) 0.5°. (b) 30°. (c) 60°. (d) 90°.


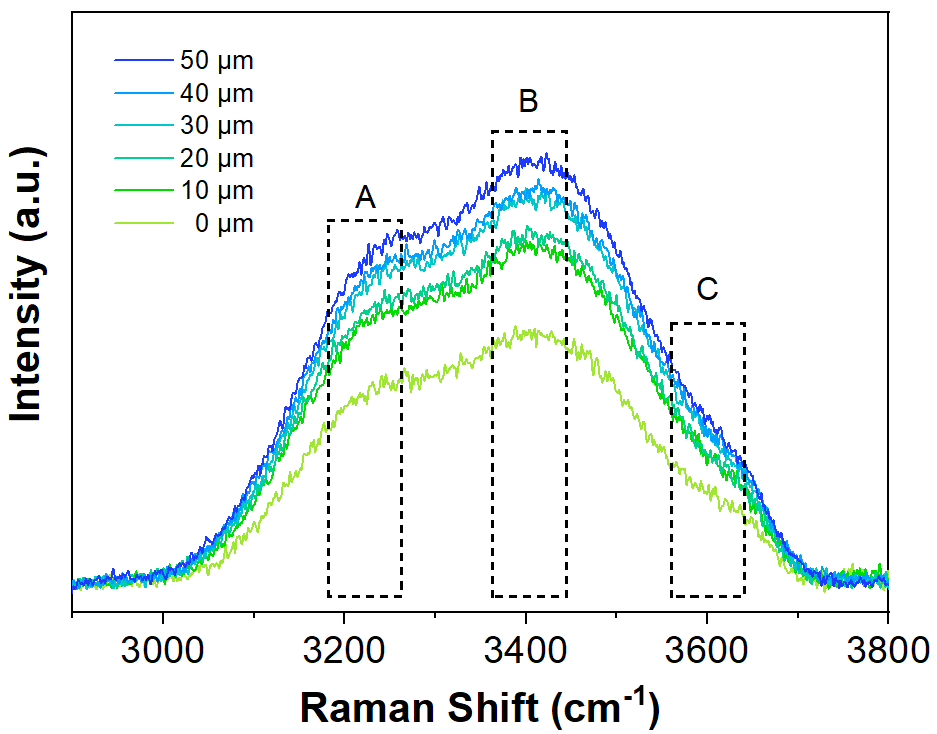


**Supplementary Fig. 8** Raman spectra of water molecules at different distances from the hydrogel surface. Bands A, B, and C correspond to water molecules with different O–H stretching vibration modes.


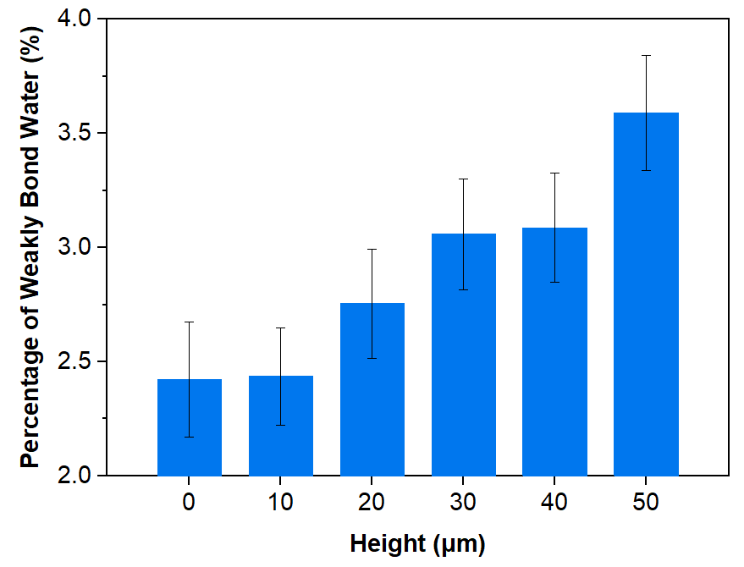


**Supplementary Fig. 9** The percentage of the free water molecules as a function of the distance from the HWT surface.


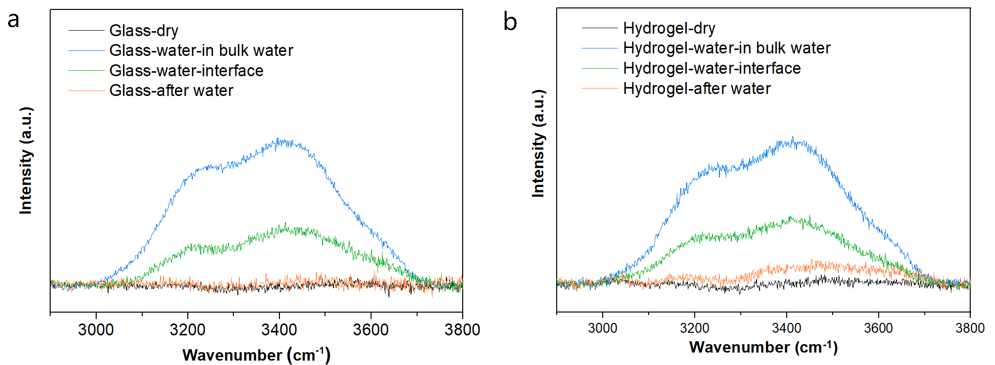


**Supplementary Fig. 10** The Raman spectrum of the water molecules on different surfaces before and after water rinsing. (a) On the glass surface. (b) On the HWT surface.


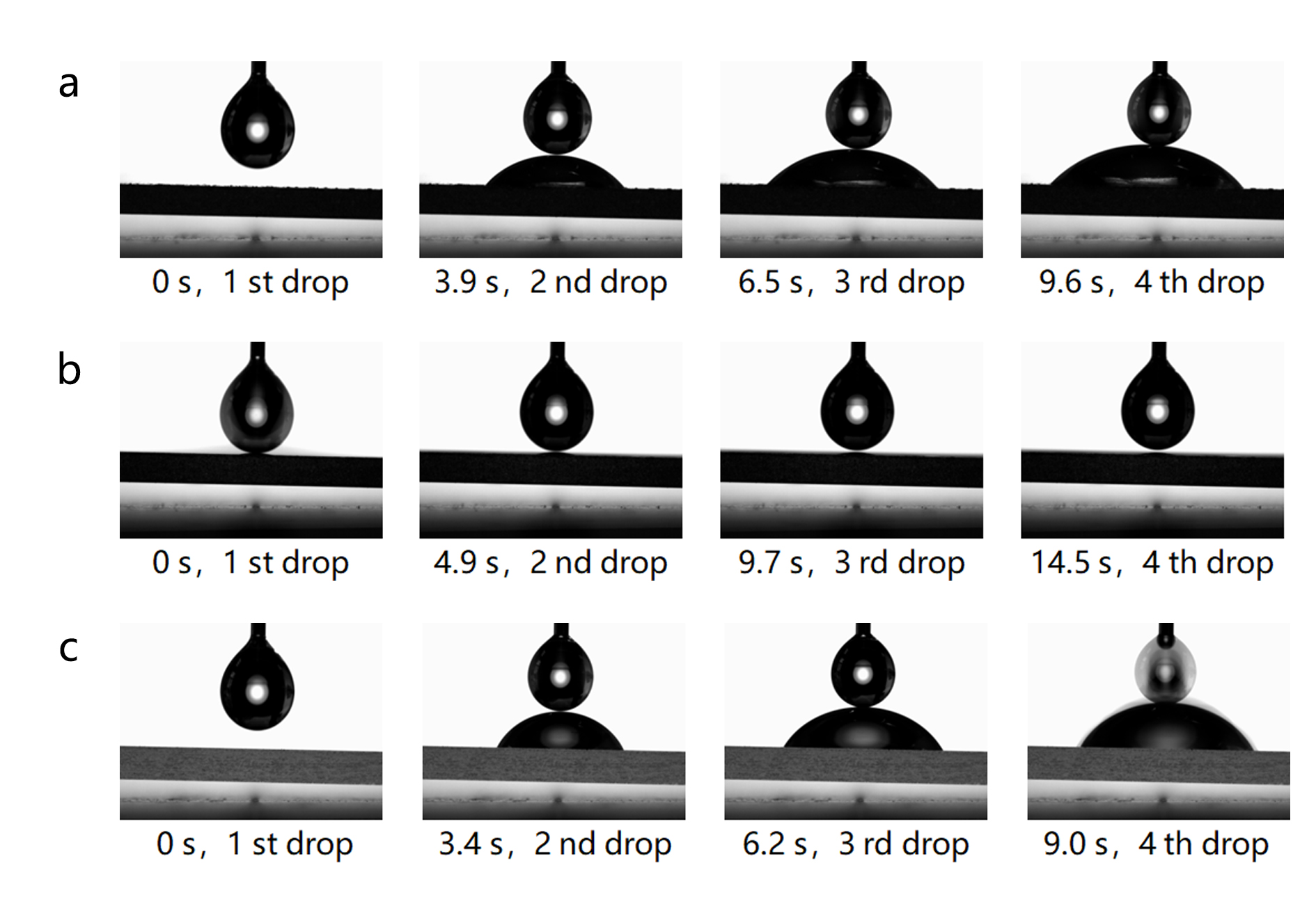


**Supplementary Fig. 11** Comparisons of the water droplet sliding on different surfaces. (a) Dry HWT. (b) Pre-wetted HWT. (c) Bare glass.


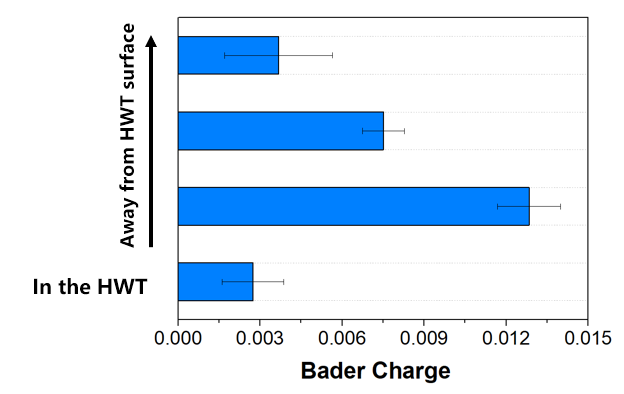


**Supplementary Fig. 12** The net charge transfer of the water molecules in the HWT and at different distances from the HWT surface.


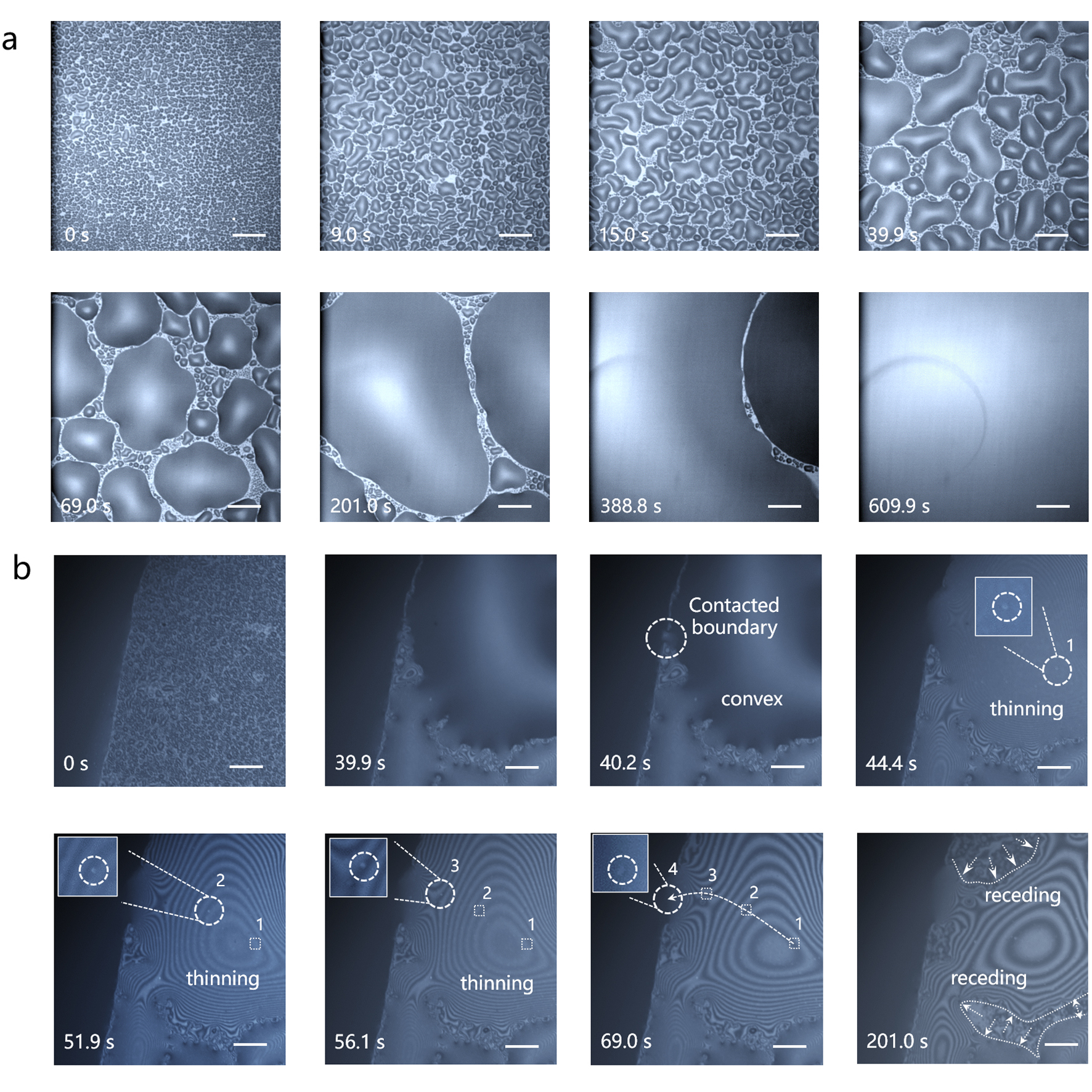


**Supplementary Fig. 13** Optical photos of the condensing process on different surfaces. (a) On the bare glass plate, the droplets formed and gradually coalesced as time went by, finally forming a water film. (b) On the HWT decorated glass, a tiny particle traced the pumping effect of the HWT, illustrating that the water droplet was pumped to the HWT and the condensing sites were re-generated (the scale bar is 100 μm).


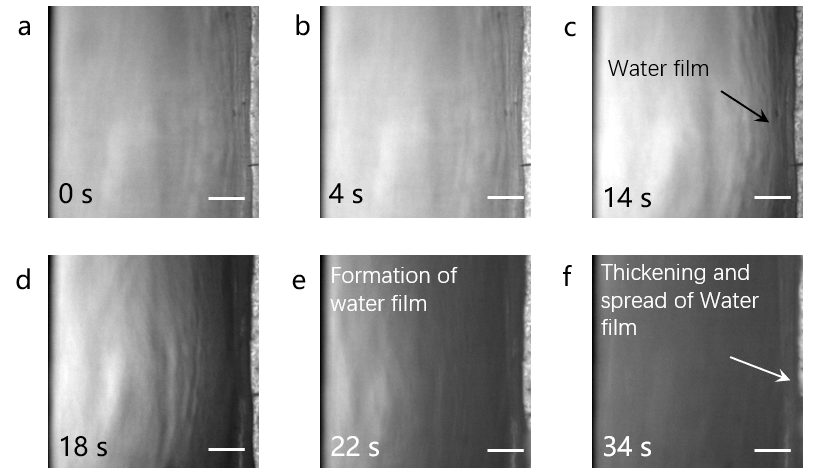


**Supplementary Fig.** **14** Optical microscope photograph of the water film-forming process during condensation. As water droplets formed and wetted the HWT surface, the reflection of the surface was changed. Water film appeared much greyer in scope, so partial coverage of water film on the HWT led to enhanced contrast. Yet as the water film spread and thickened, the whole scope became grey with the contrast decreased.


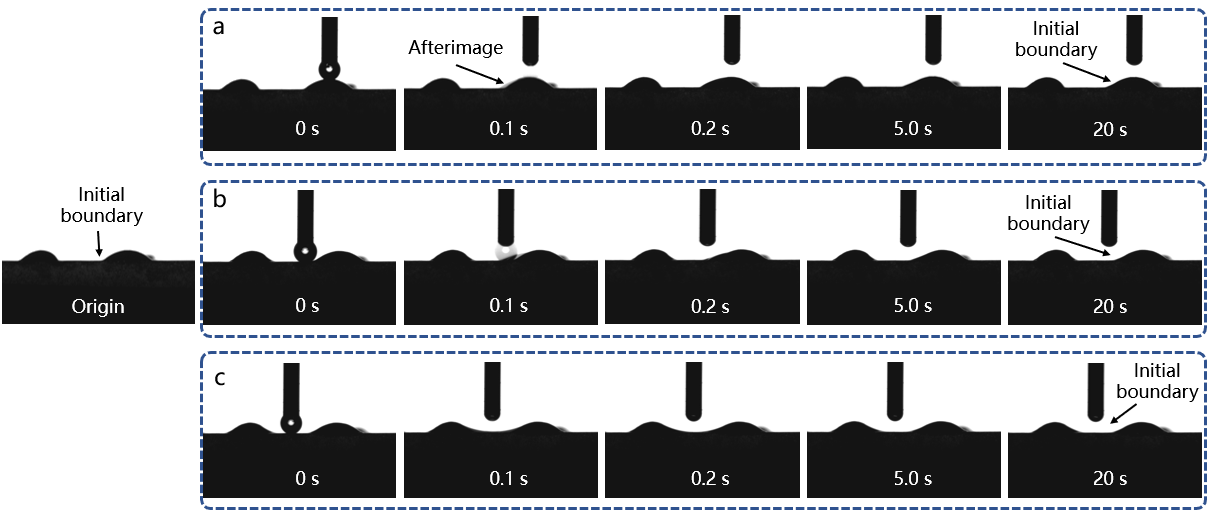


**Supplementary Fig. 15** Optical microscope photograph showing the pumping effect of the HWT. (a) The droplet was released right on top of one HWT. (b) The droplet was settled at the interface of the HWT and glass. (c) The water droplet was released right in the middle of two HWTs.


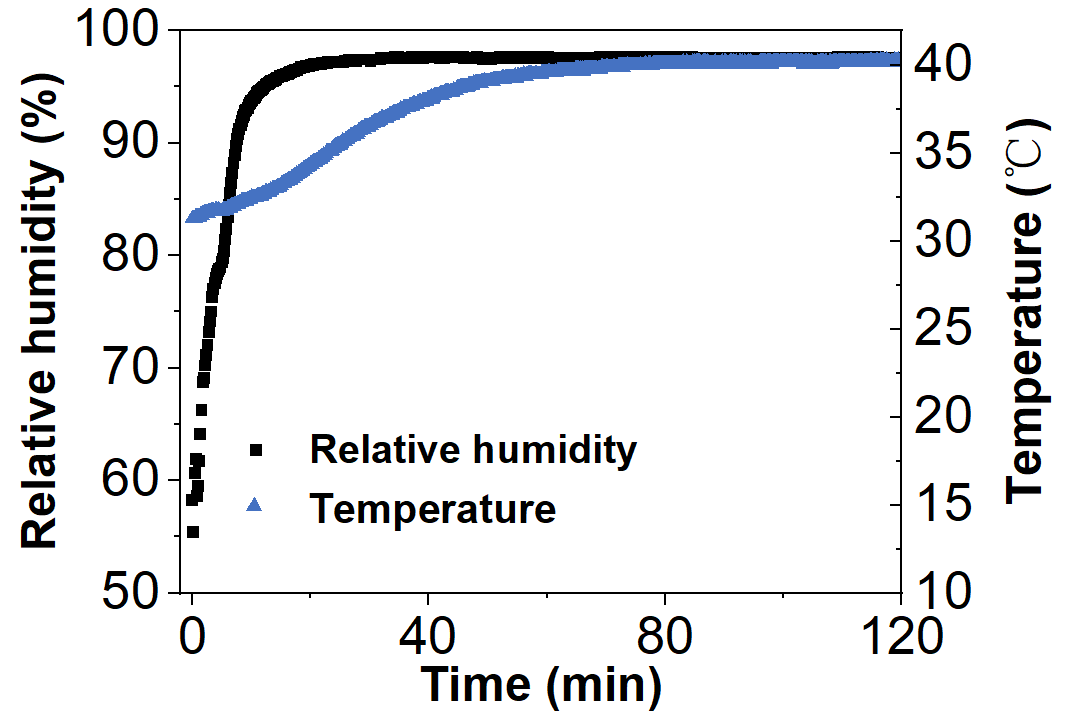


**Supplementary Fig. 16** Temperature and humidity within the condenser.


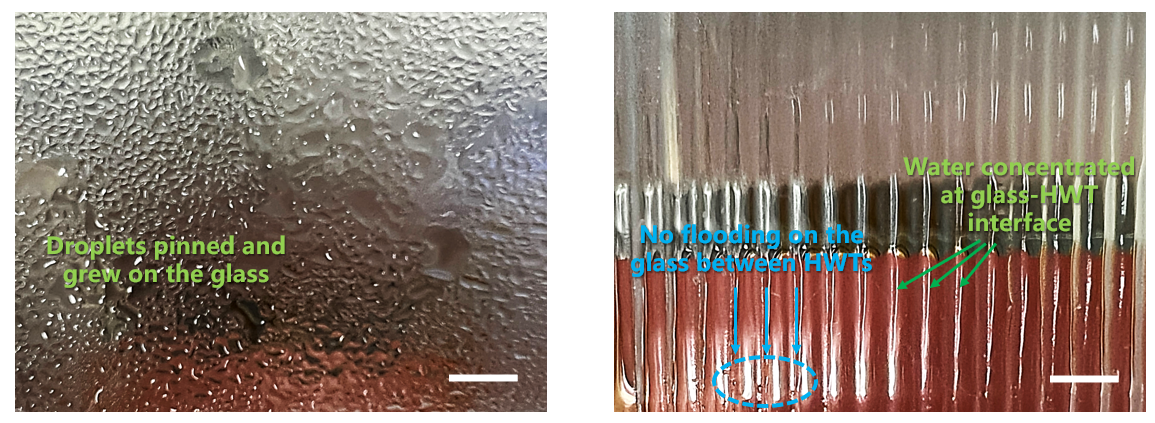


**Supplementary Fig. 17** Optical photos of the glass surface with(out) different HWTs on it during the condensation process. (a) Bare glass control. (b) HWT printed glass (scale bar is 5 mm).

**Supplementary Fig. 18** Light transmittance of the glass surface with(out) different HWTs on it during the condensation process.


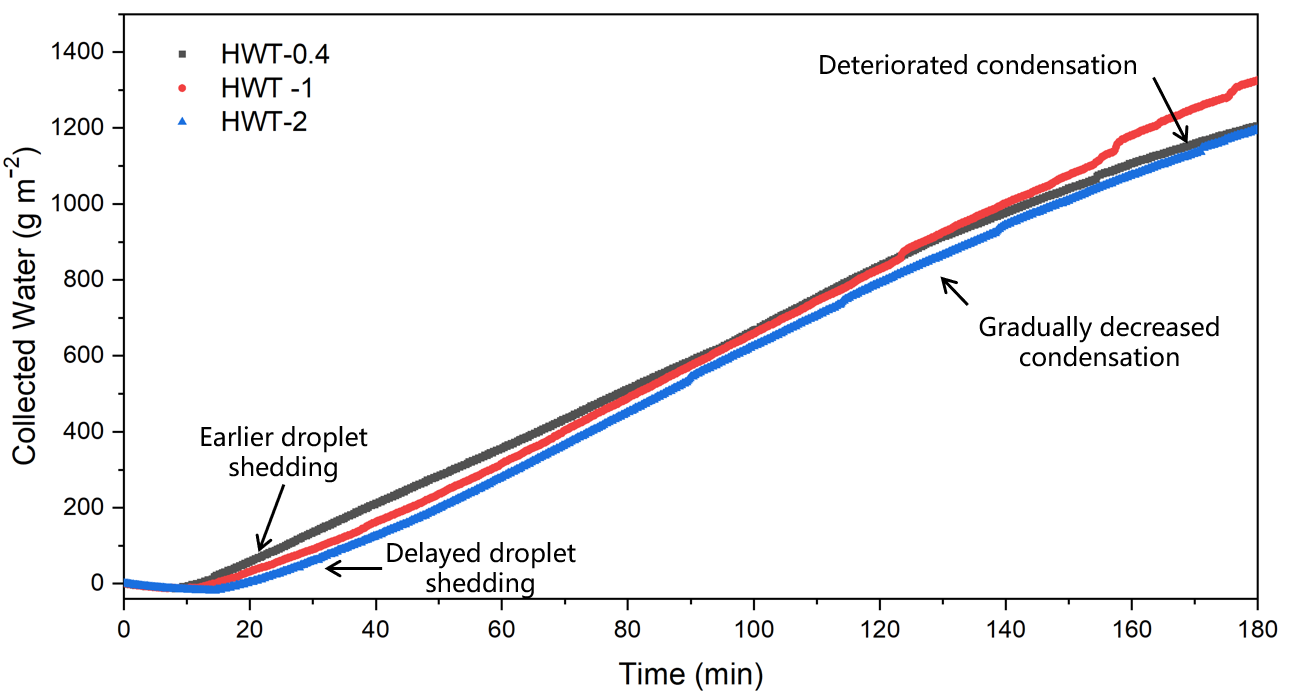


**Supplementary Fig. 19** The influence of the intervals between HWTs on the water droplet collection rates.

As shown in Supplementary Fig 20a, the conductivity of the produced water in both glass and HWT systems is ~10 μS cm^-1^, which is close to the deionized water. Based on the linear regressions, the slope of the conductivity vs. daily solar irradiance in both glass (Prob>|t|=0.30) and HWT (Prob>|t|=0.65) systems is not significant, demonstrating that the produced water qualities show no dependence on solar irradiance, so the HWT pattern is stable in sunlight. Moreover, by paired t-test, no significant difference exists between the produced water with glass and HWT pattern (Prob>|t|=0.098), further demonstrating that HWT pattern is stable when applied at the glass surface for enhanced atmospheric water harvesting (Supplementary Fig 20b).


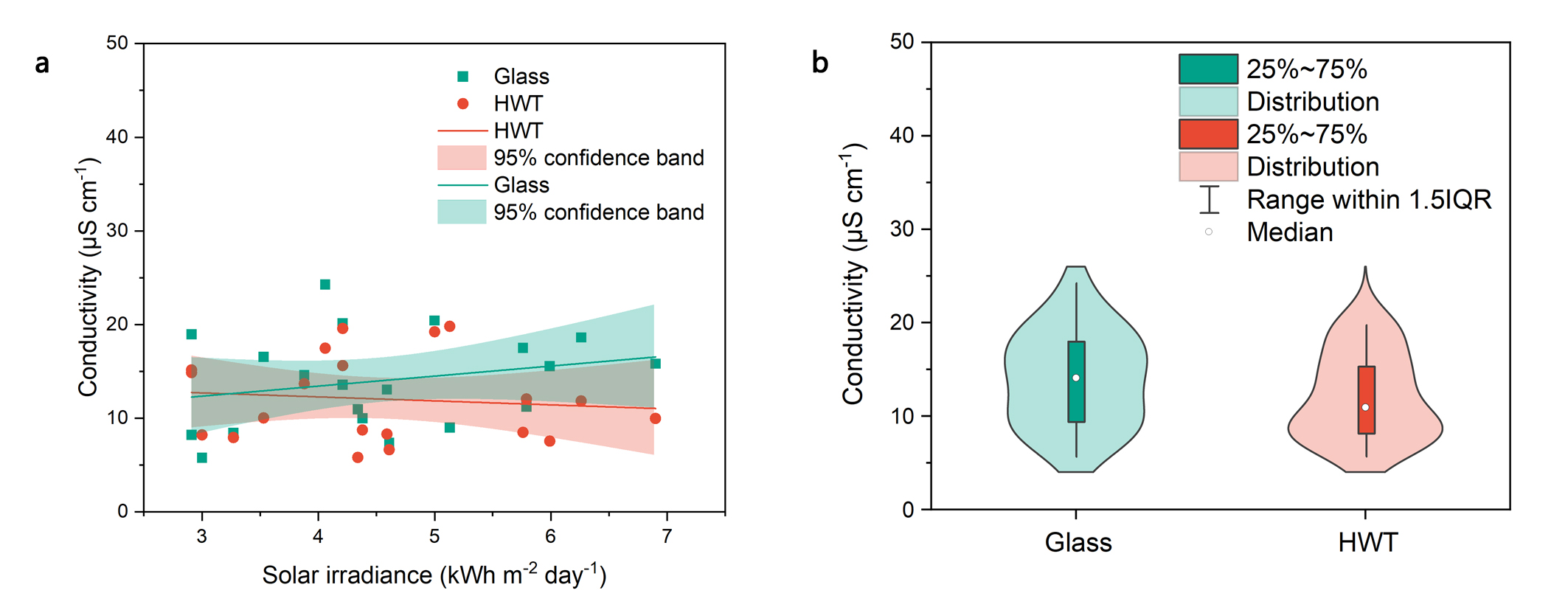


**Supplementary Fig. 20** Conductivity of the produced water with bare glass and HWT pattern. (a) Conductivity vs. daily solar irradiance. (b) The conductivity difference between the bare glass and HWT pattern.

**Supplementary Fig. 21** Ca^2+^ concentration of the collected water during outdoor experiment.


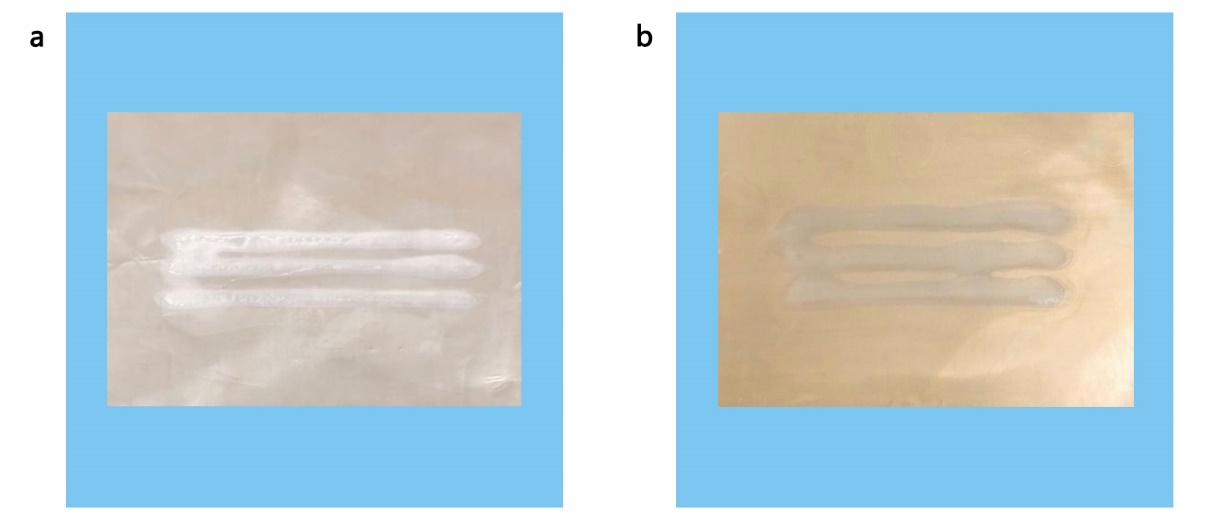


**Supplementary Fig. 22** The HWT printed on (a) aluminum and (b) copper foil, respectively.

**Table S1** The collected condensed water of outdoor solar evaporation devices.

| Solar irradiance  (kWh m^-2^) | Conditions | Collected water  (L m^-2^ day^-1^) | Ref. number in the main text |
| --- | --- | --- | --- |
| 6 (divide by 2) | In the Lab | 3.245 (divide by 2) | 43 |
| 4.81 | Rooftop test | 1.87 | 42 |
| 6.95 |  | 2.36 |  |
| 7.88 |  | 2.93 |  |
| 7 | On the sea | 5.32 (0.76 L m^-2^ h, 1 kWh m^-2^) | 44 |
| 6.47 | Floated on lake | 1.24 | 45 |
| 6.78 (integrated) | Floated on lake | 0.832 | 46 |
| 7 | Ideal maximum | 10.01 (1.43 L m^-2^ h) | 47 |

**Reference**

1. Sun H, Z Jin, C Yang, et al., COMPASS II: extended coverage for polymer and drug-like molecule databases*.* *J. Mol. Model*, 2016. **22**(2): p. 47.

2. Wang Z, Y Gong, C Jing, et al., Synthesis of dibenzotriazole derivatives bearing alkylene linkers as corrosion inhibitors for copper in sodium chloride solution: A new thought for the design of organic inhibitors*.* *Corrosion Science*, 2016. **113**: p. 64-77.

3. Ewald P P, The calculation of optical and electrostatic grid potential*.* *ANNALEN DER PHYSIK*, 1921. **64**(3): p. 253-287.

4. Andersen H C, Molecular dynamics simulations at constant pressure and/or temperature*.* *Journal of Chemical Physics*, 1980. **72**(4): p. 2384-2393.

5. Blöchl P E, Projector augmented-wave method*.* *Physical Review B*, 1994. **50**(24): p. 17953-17979.

6. Kresse G and D Joubert, From ultrasoft pseudopotentials to the projector augmented-wave method*.* *Physical Review B*, 1999. **59**(3): p. 1758-1775.

7. Hammer B, L B Hansen, and J K Nørskov, Improved adsorption energetics within density-functional theory using revised Perdew-Burke-Ernzerhof functionals*.* *Physical Review B*, 1999. **59**(11): p. 7413-7421.

8. Perdew J P, K Burke, and M Ernzerhof, Generalized Gradient Approximation Made Simple*.* *Physical Review Letters*, 1996. **77**(18): p. 3865-3868.

9. Kim S J, S G Yoon, and I K Sun, Synthesis and characteristics of interpenetrating polymer network hydrogels composed of alginate and poly(diallydimethylammonium chloride)*.* *Journal of Applied Polymer Science*, 2004. **91**(6).

10. Gao S, Y Zhu, J Wang, et al., Layer-by-Layer Construction of Cu2+/Alginate Multilayer Modified Ultrafiltration Membrane with Bioinspired Superwetting Property for High-Efficient Crude-Oil-in-Water Emulsion Separation*.* *Advanced Functional Materials*, 2018. **28**(49).

11. Zhao F, X Zhou, Y Shi, et al., Highly efficient solar vapour generation via hierarchically nanostructured gels*.* *Nature Nanotechnology*, 2018. **13**: p. 489-495.

12. Lee J, T Isobe, and M Senna, Preparation of Ultrafine Fe3O4Particles by Precipitation in the Presence of PVA at High pH*.* *Journal of Colloid and Interface Science*, 1996. **177**(2): p. 490-494.
